# Upper Arm to Upper Leg Length Ratio and Dyslipidemia: A Novel Application of a Fixed Skeletal Proportion Metric in a Nationally Representative U.S. Sample

**DOI:** 10.3390/ijerph23050662

**Published:** 2026-05-16

**Authors:** Tanvir Ahmed, Akhi Nath, Nusrat Jahan, Nargis Hoque, Mobashera Jahan, Mst Sabrina Kaniz, Shovit Dutta, Swapnil Saha, Md. Ashraful Haque, Rodney G. Bowden

**Affiliations:** 1 Department of Public Health, Baylor University, Waco, TX 76798, USA; tanvir_ahmed1@baylor.edu; 2 School of Information Technology Management, Toronto Metropolitan University, Toronto, ON M5B 2K3, Canada; akhi.nath@torontomu.ca; 3 Department of Public Health, North South University, Dhaka 1229, Bangladesh; nusrat.jahan30@northsouth.edu (N.J.); mobashera.jahan@northsouth.edu (M.J.); 4 Department of Public Health, National University of Preventive and Social Medicine (NIPSOM), Dhaka 1212, Bangladesh; nargishoque11@gmail.com; 5 Department of Public Health, Macquarie University, Sydney 2109, Australia; mstsabrina.kaniz@students.mq.edu.au; 6 Department of Pediatrics, BGC Trust Medical College Hospital, Chittagong 4381, Bangladesh; drshovit@bgctmc.ac.bd; 7 Department of Computer Science, Baylor University, Waco, TX 76798, USA; swapnilsaha69@gmail.com; 8 Maternal and Child Health Division, International Centre for Diarrhoeal Disease Research, Dhaka 1212, Bangladesh; ashraful.haque3@icddrb.org; 9 Medical Sciences and Department of Public Health, Baylor University, Waco, TX 76798, USA

**Keywords:** dyslipidemia, upper arm-upper leg length ratio, skeletal proportions, cardiometabolic risk, anthropometry, NHANES, cardiovascular disease

## Abstract

**Highlights:**

**Public health relevance—How does this work relate to a public health issue?**

- Dyslipidemia is a major, highly prevalent risk factor for cardiovascular disease, which remains a leading cause of morbidity and mortality in the U.S. adult population; this study examines, for the first time in a large nationally representative U.S. sample, whether the upper arm-to-upper leg length ratio (UA/UL), a fixed skeletal proportion metric, is associated with dyslipidemia, extending prior work on body proportion metrics and metabolic risk to this lipid outcome.
- The study evaluates a fixed skeletal proportion (upper arm–to–upper leg length ratio) using nationally representative NHANES data, linking early-life developmental body structure to adult cardiometabolic health outcomes relevant to population health.

**Public health significance—Why is this work of significance to public health?**

- Unlike conventional anthropometric measures that change over time, the UA/UL ratio reflects stable skeletal development and may capture early-life biological and environmental influences associated with dyslipidemia risk, particularly among younger adults, with associations in sex-stratified and age-stratified models attenuating after full adjustment for modifiable factors.
- Identifying supplementary, low-cost, and easily measurable markers of dyslipidemia risk has public health value for improving early risk stratification beyond body mass index (BMI) and waist-based measures, especially in settings where longitudinal data are limited.

**Public health implications—What are the key implications or messages for practitioners, policy makers and/or researchers in public health?**

- For practitioners: Fixed anthropometric measures such as the UA/UL ratio may warrant investigation as supplementary markers to support lipid risk assessment; however, clinical application requires prospective validation and establishment of standardized cutpoints before any recommendation can be made.
- For policy makers and researchers: Findings support further longitudinal research to determine whether skeletal proportion metrics such as the UA/UL ratio independently predict cardiometabolic outcomes; premature incorporation into prevention strategies is not yet warranted without prospective validation.

**Abstract:**

Conventional anthropometric measures used to predict dyslipidemia, such as body mass index and waist circumference, vary over time and may not fully capture early-life influences on metabolic risk. Fixed skeletal proportions, including limb length ratios, remain stable after physical maturity and may reflect developmental exposures relevant to lipid metabolism. This study examined the association between the upper arm–to–upper leg length ratio (UA/UL), a fixed skeletal proportion metric with established links to diabetes risk and dyslipidemia; this represents an application not previously reported in a nationally representative U.S. population. We conducted a cross-sectional analysis of adults aged ≥20 years using data from the National Health and Nutrition Examination Survey (NHANES) 2017–March 2020 (*n* = 7569). The UA/UL ratio was calculated from standardized upper arm and upper leg length measurements and categorized into quartiles based on the weighted sample distribution. Dyslipidemia was defined according to National Cholesterol Education Program Adult Treatment Panel III criteria or current lipid-lowering medication use. Survey-weighted logistic regression models were used to estimate odds ratios (ORs) and 95% confidence intervals (CIs) across progressively adjusted models. Dyslipidemia prevalence increased across UA/UL quartiles (58.4% in Q1 to 81.3% in Q4; *p* < 0.001). In unadjusted analyses, individuals in the highest UA/UL quartile had greater odds of dyslipidemia compared with the lowest quartile (OR 3.10, 95% CI 2.49–3.86). Associations remained significant after adjustment for demographic factors and for anthropometric measures considered separately. However, the association was attenuated and no longer statistically significant in fully adjusted models that included demographics, adiposity measures, hypertension, and diabetes. In sex-stratified analyses, the association was attenuated and no longer statistically significant in either sex after full adjustment; formal interaction testing confirmed no significant effect modification by sex (*p*-for-interaction = 0.943).

## 1. Introduction

Dyslipidemia is a clinical condition characterized by abnormal levels of blood lipids such as total cholesterol (TC), triglycerides (TG), low-density lipoprotein cholesterol (LDL-C), and high-density lipoprotein cholesterol (HDL-C) [1]. It is considered as one of the primary modifiable risk factors for cardiovascular disease [2] and remains a key contributor to cardiovascular morbidity and mortality globally [3]. Findings from the Global Burden of Disease 2019 study indicate that elevated LDL-C contributed to an estimated 4.4 million deaths and 98 million disability-adjusted life years worldwide [4]. Despite advances in prevention and treatment, dyslipidemia continues to be a major public health concern in the United States [5]. According to the 2025 AHA Heart Disease and Stroke Statistics Update, approximately 25.5% of U.S. adults had LDL-C levels ≥130 mg/dL and 16.9% had low HDL-C, highlighting the continued burden of lipid abnormalities [6]. Identifying individuals at risk at an early stage is therefore essential to reducing the long-term cardiovascular disease burden associated with lipid disorders.

Body mass index (BMI) has long been used as the most common anthropometric measure for predicting obesity-related health risks, including dyslipidemia [7]. Even though BMI has been used extensively, it has failed to predict dyslipidemia with higher accuracy [8]. Newer anthropometric indices such as waist circumference (WC), hip circumference (HC), waist-to-hip ratio (WHR), and waist-to-height ratio (WtHR) have been introduced to enhance the prediction of dyslipidemia [9]. Among these indices, WC and WtHR have the strongest associations with dyslipidemia, particularly elevated TG and reduced HDL-C [10]. WtHR is generally regarded as more reliable, as it takes into account the differences in height and demonstrates consistent predictive value across different ethnic and sex groups [11].

Although these anthropometric measures provide improved predictive ability, they are not fixed parameters and can vary over time in response to age, physical activity, and dietary habits [12]. The changing nature of all the commonly used anthropometric measurements initiates the increasing interest in exploring fixed anthropometric measurements that remain stable after physical maturity and could serve as lifelong predictors of dyslipidemia.

One fixed skeletal anthropometric measure in adults is the upper arm–to–upper leg ratio (UA/UL), defined as the length of the upper arm divided by the length of the upper leg; conceptually, it is analogous to the humero-femoral index, which is based on direct bone lengths [13]. Upper arm length is measured as the distance between the acromion process (bony tip of the shoulder) and the olecranon process (tip of the elbow), while upper leg length is measured from the greater trochanter (top of the femur near the hip) to the lateral condyle of the tibia (outer knee) [14]. This ratio reflects skeletal proportions rather than adiposity and can be easily obtained through standardized anthropometric assessment [15]. Because limb growth generally stops by around 20 years of age, this ratio remains relatively constant in adulthood and may capture developmental influences on metabolic health [16].

Previous research has reported that body proportions, particularly shorter leg length relative to trunk length, are associated with insulin resistance and adverse lipid profiles [15]. A recently published study reported that the upper arm to upper leg ratio was significantly associated with type 2 diabetes. Individuals with higher ratios showed a greater risk of developing diabetes even after adjusting for BMI and WC [13]. The upper arm-to-upper leg length ratio (UA/UL) is a fixed skeletal proportion that can serve as a proxy for regional body composition and fat distribution established early in life, consistent with evidence that limb lengths reflect early developmental environments and later metabolic risk [17]. Shorter relative leg length captured by a higher UA/UL ratio has repeatedly been linked to insulin resistance and adverse lipid profiles, suggesting that limb proportions index developmental exposures (e.g., early life nutrition or hormonal milieu) that program long-term cardiometabolic risk, aligning with findings that shorter limb segments predict diabetes independent of adiposity [18] Early-life nutritional status is a principal determinant of limb growth: protein-energy undernutrition during the first two years of life preferentially restricts lower-limb elongation relative to upper-limb growth, producing persistently higher arm-to-leg length ratios in adulthood that may therefore serve as a fixed anthropometric record of early nutritional deprivation [15,16]. Beyond nutrition, a range of prenatal and postnatal developmental exposures, including intrauterine growth restriction, low birth weight, maternal undernutrition during pregnancy, recurrent childhood infection, and socioeconomic deprivation, have been independently associated with shorter adult leg length and altered skeletal proportions, consistent with the concept that the early developmental environment shapes fixed body composition patterns that persist throughout the life course [18]. These observations align with the Developmental Origins of Health and Disease (DOHaD) framework [16], which proposes that suboptimal early-life environments program lasting metabolic adaptations, including impaired insulin signaling and dysregulated lipid metabolism, that manifest as elevated cardiometabolic risk in adulthood, offering a plausible biological pathway through which a higher UA/UL ratio may mark long-term susceptibility to dyslipidemia.

Prior published findings suggest that shorter legs relative to trunk are associated with higher TG, lower HDL-C, and greater insulin resistance independent of overall adiposity, supporting the premise that upper-body predominance reflects a more atherogenic phenotype [19]. Analogous skeletal metrics such as the humero-femoral index have been associated with type 2 diabetes risk even after adjusting for BMI and WC, reinforcing the idea that fixed proportions may mark metabolic susceptibility beyond contemporaneous adiposity [20].

Mechanistically, if relatively shorter legs indicate constrained lower-body (gluteo-femoral) fat-storage capacity, excess energy may be redirected to upper-body and ectopic depots that are more lipolytically active, promoting hepatic very low density lipoprotein cholesterol (VLDL) overproduction, lower HDL-C, and hypertriglyceridemia via insulin-resistant pathways, patterns consistent with the metabolic implications of altered limb proportions [21]. Notably, sex differences in adipose biology, with women typically exhibiting greater peripheral subcutaneous storage, offer a plausible basis for the stronger associations observed in females, as disproportion favoring upper-body mass may carry greater metabolic penalties when lower-body buffering capacity is limited [13]. Taken together, the UA/UL ratio may integrate developmental programming of limb growth with adult regional adiposity patterns, thereby predicting dyslipidemia through established insulin-resistance and lipid-metabolism pathways, even when standard adiposity markers are accounted for in studies [22,23]. These findings suggest that fixed anthropometric measurements reflecting skeletal proportion may serve as useful predictors of metabolic diseases such as dyslipidemia.

Therefore, the present study aims to examine, for the first time in a nationally representative U.S. sample, the association between the UA/UL ratio, a fixed skeletal proportion measure previously linked to diabetes risk, and dyslipidemia. This investigation uses data from the National Health and Nutrition Examination Survey (NHANES) 2017–March 2020, which includes standardized measurements of upper arm and upper leg length. No prior study has examined the association between the UA/UL ratio and dyslipidemia in a nationally representative U.S. population. Demonstrating such an association would extend existing evidence linking body proportion metrics to cardiometabolic outcomes and offer insight into how fixed skeletal structure, as a proxy for early developmental exposures, may be associated with adult lipid metabolism.

## 2. Methods

### 2.1. Study Design and Data Source

The National Health and Nutrition Examination Survey (NHANES) data from 2017 to March 2020 were used in this study to examine the association between the upper arm-to-upper leg length ratio and dyslipidemia. The data were collected through a cross-sectional survey conducted by the National Center for Health Statistics (NCHS), which provided a representative sample of the civilian, non-institutionalized U.S. population and included demographic, health, and nutrition information, with detailed laboratory data on lipid profiles, including LDL-C, TC, TG, and HDL-C. The NCHS Institutional Review Board obtained ethical approval for NHANES, and informed consent was obtained from all participants in the data collection process. Detailed information about the survey design methods and data collection procedures of NHANES 2017–March 2020 can be found at https://wwwn.cdc.gov/nchs/nhanes/continuousnhanes/default.aspx?Cycle=2017-2020 (accessed on 24 March 2026).

### 2.2. Study Population

**Figure 1:**
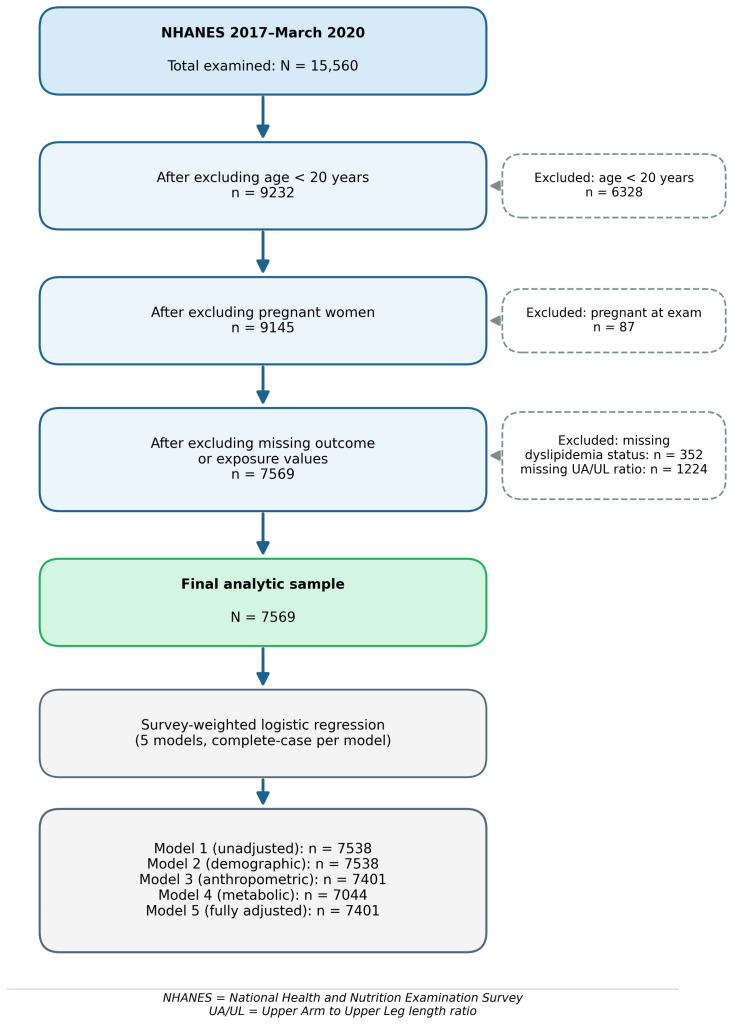
Participant selection flow diagram. NHANES 2017–March 2020 (*N* = 15,560 total examined; *N* = 7569 final analytic sample). UA/UL = upper arm to upper leg length ratio.

A total of 15,560 participants were examined in the National Health and Nutrition Examination Survey (NHANES) 2017–March 2020 pre-pandemic cycles. Participants were excluded in four sequential steps. First, all individuals aged under 20 years were excluded (*n* = 6328) because skeletal bone elongation continues until approximately this age, and the UA/UL ratio is only biologically meaningful as a fixed adult anthropometric measure once bone growth is complete [13]. This left 9232 adults. Second, women who were pregnant at the time of the examination (NHANES variable RIDEXPRG = 1) were excluded (*n* = 87), as pregnancy alters body composition and lipid profiles in ways that would confound both the exposure and outcome measurements. This left 9145 participants. Third, participants with missing dyslipidemia classification or missing UA/UL ratio—or both—were excluded (*n* = 1576 total: 352 missing dyslipidemia status only, 653 missing UA/UL ratio only, and 571 missing both). Dyslipidemia classification required non-missing values for total cholesterol (LBXSCH), HDL-C (LBDHDD), triglycerides (LBXSTR), and calculated LDL-C, itself derived using the Friedewald equation (LDL = total cholesterol − HDL-C − [triglycerides ÷ 5]), which is only applicable when triglycerides are below 400 mg/dL; participants with triglycerides at or above this threshold were assigned a missing LDL-C value. The UA/UL ratio required non-missing values for both BMXARML and BMXLEG. The final analytic dataset consisted of 7569 participants (48.6% of the original NHANES 2017–March 2020 sample; Figure 1). Across all regression models, a complete-case approach was used; participants with missing data on any model covariate were excluded by the model fitting procedure. Analytic sample sizes therefore varied across models: *n* = 7538 in the unadjusted model, *n* = 7401 in models additionally adjusting for diabetes and anthropometric covariates, and *n* = 7044 in the model further including hypertension status (which had the highest covariate missingness, *n* = 372 missing, as reported in Table 1). Given that 7991 participants (51.4% of the original sample) were excluded overall, and that those excluded may systematically differ from those included in ways relevant to the study question (e.g., younger age, missing lipid measurements more common in healthier individuals), a comparison of baseline characteristics between included and excluded participants is presented in Supplementary Table S1 to allow readers to assess the direction and potential magnitude of selection bias.

### 2.3. Variable Definitions

#### 2.3.1. Dyslipidemia

Dyslipidemia was defined according to the National Cholesterol Education Program Adult Treatment Panel III (NCEP ATP III) criteria as the presence of one or more abnormal lipid parameters or current use of lipid-lowering medication [17]. Participants were classified as having dyslipidemia if they met any one or more of the following criteria, each applied independently using OR logic (meeting any single criterion was sufficient for classification; participants were not required to meet multiple thresholds simultaneously):

- Low-density lipoprotein cholesterol (LDL-C) ≥ 130 mg/dL
- Total cholesterol ≥ 200 mg/dL
- Triglycerides ≥ 150 mg/dL
- High-density lipoprotein cholesterol (HDL-C) < 40 mg/dL for men or <50 mg/dL for women
- Current use of cholesterol-lowering medication

LDL-C was not directly measured in NHANES but was estimated using the Friedewald equation: LDL-C = total cholesterol − HDL-C − (triglycerides ÷ 5), all in mg/dL. This calculation was applied only to participants with triglycerides below 400 mg/dL; participants with triglycerides at or above this threshold were assigned a missing LDL-C value, as the Friedewald formula is not valid at high triglyceride concentrations. Current use of cholesterol-lowering medication was ascertained from NHANES questionnaire variable BPQ090D (self-reported current use of prescribed cholesterol-lowering medication). In the absence of a directly measured LDL-C variable, this operationalization using the Friedewald-derived LDL-C, alongside the three other lipid parameters and medication use, constitutes the complete dyslipidemia definition applied in this study.

To characterize the individual lipid components contributing to the composite dyslipidemia outcome, the survey-weighted prevalence of each criterion (total cholesterol ≥ 200 mg/dL; LDL-C ≥ 130 mg/dL; triglycerides ≥ 150 mg/dL; sex-specific low HDL-C; current lipid-lowering medication use) was computed overall and by UA/UL quartile using PROC SURVEYFREQ with Rao–Scott chi-square tests. Additionally, survey-adjusted logistic regression models (equivalent to Model 2 covariates) were run separately for each component as the binary outcome to determine which lipid fraction drove the observed composite association. Results of these component-specific analyses are presented in Supplementary Table S2. Two components showed significant associations with UA/UL quartile after demographic adjustment: hypertriglyceridemia (TG ≥ 150 mg/dL; *p* < 0.001) and low HDL-C (*p* < 0.001). Total cholesterol ≥ 200 mg/dL (*p* = 0.369) and LDL-C ≥ 130 mg/dL (*p* = 0.601) were not significantly associated with UA/UL quartile.

#### 2.3.2. Upper Arm-to-Upper Leg Length Ratio and Quartile

The UA/UL is a skeletal proportion traditionally defined as the ratio of humerus length to femur length [13]. It is calculated from the upper arm length and the upper leg length, two distinct body measures captured by NHANES during the collection of examination data, along with other body measures. These body measures were collected by trained health technicians in the Mobile Examination Centers. The upper arm and leg measurements were mostly done on the right-side limbs, except for exceptional situations where the participant had certain physical or medical conditions (e.g., amputation, medical appliances such as casts) that prevented measurements on the right-side limbs [24]. In instances where right-side limb measurements were unable to be collected, measurements were made on the left side limbs. Both measures were reported in centimeters (cm). Although the UA/UL ratio is conceptually analogous to the humero-femoral index—which is based on direct osteometric measurements of the humerus and femur—the NHANES measurements used here assess surface anthropometry of the upper arm (acromion to olecranon process) and upper leg (greater trochanter to lateral condyle of the tibia). These measurements, therefore, capture overlying soft tissue in addition to the underlying bone and should be understood as a soft-tissue anthropometric proxy for skeletal proportions rather than a direct osteometric measure. This distinction is relevant to interpreting results: the UA/UL ratio is expected to be stable in adulthood and largely driven by underlying skeletal structure, but may differ from direct bone-length measurements in individuals with markedly asymmetric soft-tissue distributions. The ratio is calculated as upper arm length (cm) divided by upper leg length (cm).

Because no clinically established cutoffs exist for the UA/UL ratio, quartiles were defined empirically based on the weighted distribution of the study population, consistent with prior NHANES-based anthropometric analyses. Therefore, for better interpretability of data, we further categorized the upper arm length to upper leg length ratio into four quartiles based on the empirical weighted distribution of the study population. The precise empirical cutpoints derived from PROC UNIVARIATE on the survey-weighted distribution were: Q1 < 0.898345; Q2 ≥ 0.898345 to <0.942408; Q3 ≥ 0.942408 to <0.992806; Q4 ≥ 0.992806.

#### 2.3.3. Non-Modifiable Factors

As non-modifiable factors that can influence dyslipidemia, we included age, sex, and race/ethnicity as covariates [25]. In the NHANES data, Age is reported in years for adult participants. We have further categorized the “Age” into four age groups: 20–34 years, 35–50 years, 51–65 years, and >65 years. Age was categorized into four groups to reflect adult life stages and to support descriptive and stratified analyses. This grouping was also supported empirically by the marked increase in dyslipidemia prevalence across age categories in the study population.

Sex was categorized as “Male” and “Female”. Race classified as Mexican American, Non-Hispanic White, Non-Hispanic Black, Other Hispanic, and Other Race, including multi-racial, based on NHANES classifications. 

#### 2.3.4. Modifiable Factors

Based on previous literature [17], we have included the following additional covariates, as these are established modifiable risk factors for dyslipidemia. BMI was entered into regression models as a continuous variable (kg/m^2^). For descriptive purposes, BMI was categorized as: underweight (<18.5), normal weight (18.5–24.99), overweight (25–29.99), and obesity (≥30) [26]. Waist circumference (WC) was reported in cm in the NHANES data. WC was entered as a continuous variable in regression models. For descriptive purposes, WC was categorized using WHO sex-specific thresholds: normal (<94 cm in men or <80 cm in women), increased risk (94 to <102 cm in men or 80 to <88 cm in women), and substantially increased risk (≥102 cm in men or ≥88 cm in women) [17]. Hip circumference (HC) was also entered as a continuous variable in regression models. Hypertension status was ascertained using NHANES blood pressure measurements and self-reported physician diagnosis, consistent with standard clinical thresholds for hypertension classification [27]. Diabetes status was defined as current insulin use (DIQ050 = 1), current use of oral diabetes medication (DIQ070 = 1), HbA1c ≥ 6.5% (LBXGH), or blood glucose ≥ 126 mg/dL (LBXGLU or LBXSGL), consistent with American Diabetes Association diagnostic criteria [28]. Participants with missing values for all three laboratory measures were assigned a missing diabetes classification. Both hypertension and diabetes were treated as binary covariates (yes/no) in all regression models.

## 3. Results

### 3.1. Participant Characteristics

The survey weighted characteristics of the study population, consisting of U.S. adults aged 20 years and older, are presented by dyslipidemia status in Table 1. Dyslipidemia prevalence increased across age groups, from 50.8% among adults aged 20–34 years to 86.2% among those aged ≥65 years (*p* < 0.001). Prevalence did not differ by sex (*p* = 0.68) but differed across race and ethnicity categories, with Non-Hispanic White adults showing the highest prevalence (72.4%) and Non-Hispanic Black adults the lowest (61.1%) (*p* < 0.001). Dyslipidemia prevalence also differed across BMI categories and WC risk categories and was higher among adults with hypertension and diabetes (all *p* < 0.001). Dyslipidemia prevalence increased across UA/UL ratio quartiles, from 58.4% in Q1 to 81.3% in Q4 (*p* < 0.001).

Table 2 presents participant characteristics by UA/UL quartiles. The distribution of UA/UL quartiles differed by age and sex, with a greater share of older adults and a higher proportion of females in Q4 (both *p* < 0.001). Participants in Q4 also had higher prevalence of obesity, central obesity, hypertension, and diabetes (all *p* < 0.001). For example, 50.4% of participants with diabetes were in Q4 compared with 8.1% in Q1, and 38.2% of participants in the obese WC category were in Q4 compared with 13.5% in Q1.

### 3.2. Association Between UA/UL Ratio and Dyslipidemia

The association between UA/UL quartiles and the odds of dyslipidemia was evaluated using progressively adjusted weighted logistic regression models in Table 3: All analyses accounted for the complex multistage probability sampling design of NHANES using the R survey package (svydesign function). The survey design object was specified as: primary sampling units (id = ~SDMVPSU), sampling strata (strata = ~SDMVSTRA), and examination weights (weights = ~WTMECPRP), with nest = TRUE to reflect the nested structure of PSUs within strata. The weight variable WTMECPRP is the mobile examination center (MEC) combined pre-pandemic weight released by NCHS specifically for the 2017–March 2020 dataset; this weight appropriately accounts for the truncated 2019–2020 data collection period and should be used in place of the standard 2-year MEC weight (WTMEC2YR) for this combined cycle. Logistic regression models were fitted using the svyglm function with a quasibinomial family, which is appropriate for binary outcomes under complex survey designs and accommodates mild overdispersion (dispersion parameters ranged from 1.09 to 1.16 across models). Standard errors were estimated using the linearization method implemented in the survey package. Odds ratios (ORs) and 95% confidence intervals (CIs) were obtained by exponentiating model coefficients and their confidence limits. All analyses were performed in R (version 4.4.3).

- Model 1 (Crude): In the unadjusted model, higher UA/UL quartiles were significantly associated with increased odds of dyslipidemia. Individuals in Q4 had significantly greater odds of dyslipidemia compared to those in Q1 (OR 3.10, 95% CI 2.49–3.86; *p* < 0.001).
- Model 2 (Non-modifiable Factors): After adjusting for sex, race, and age group, the association remained significant for Q3 (OR 1.37, 95% CI 1.05–1.78; *p* = 0.020) and Q4 (OR 1.72, 95% CI 1.27–2.33; *p* < 0.001).
- Model 3 (Anthropometric Factors): Adjustment for BMI, WC, and HC maintained a significant association for Q3 (OR 1.56, 95% CI 1.23–1.97; *p* < 0.001) and Q4 (OR 1.97, 95% CI 1.58–2.46; *p* < 0.001). This suggests that the UA/UL ratio remains associated with dyslipidemia after controlling for conventional adiposity markers, though this model does not yet include demographic confounders.
- Model 4 (Metabolic Factors): When adjusted for hypertension and diabetes, Q4 continued to show a significant association (OR 2.01, 95% CI 1.53–2.64; *p* < 0.001).
- Model 5 (Final Full Model): In the final model, adjusting for all non-modifiable, anthropometric, and metabolic factors, the independent association between UA/UL quartiles and dyslipidemia was attenuated and no longer reached statistical significance (Q4 OR 0.92, 95% CI 0.65–1.32; *p* = 0.654).

To characterize which lipid component drove the composite dyslipidemia outcome, survey-weighted logistic regression models equivalent to Model 2 were run separately for each component as a binary outcome (Supplementary Table S2). The UA/UL ratio was significantly associated with hypertriglyceridemia (TG ≥ 150 mg/dL; *p* < 0.001; Q4 OR 1.84, 95% CI 1.31–2.60) and low HDL-C (*p* < 0.001; Q4 OR 1.97, 95% CI 1.58–2.46). By contrast, neither elevated total cholesterol (*p* = 0.369) nor elevated LDL-C (*p* = 0.601) was significantly associated with UA/UL quartile after demographic adjustment. Lipid-lowering medication use showed a significant dose–response pattern (Q4 OR 2.02, 95% CI 1.42–2.88; *p* = 0.004), though this association is likely substantially confounded by age. These component-level results indicate that the UA/UL–dyslipidemia association is driven primarily by the TG/HDL-C axis, consistent with an insulin resistance-driven metabolic phenotype rather than an LDL-driven atherogenic pattern.

### 3.3. Subpopulation Analysis

Sex-stratified analysis (Table 4) showed that higher UA/UL quartiles were significantly associated with greater odds of dyslipidemia in unadjusted models among both females (Q4 OR 3.07, 95% CI 2.39–3.95; *p* < 0.001) and males (Q4 OR 3.19, 95% CI 2.29–4.46; *p* < 0.001). After adjustment for modifiable factors and chronic diseases, the association was attenuated and no longer statistically significant in either sex (females: Q4 OR 1.19, 95% CI 0.86–1.66, *p* = 0.307; males: Q4 OR 1.42, 95% CI 0.96–2.10, *p* = 0.091). Formal tests of the UA/UL quartile × sex interaction were conducted in the unadjusted (*p*-for-interaction = 0.984) and demographic-adjusted models (*p*-for-interaction = 0.943). Both were strongly non-significant, indicating that sex does not significantly modify the UA/UL ratio–dyslipidemia association. The observed sex difference in stratified estimates is therefore consistent with chance variation from multiple subgroup comparisons rather than genuine effect modification, and should be interpreted as exploratory only.

Age-specific analysis (Table 5) showed that the association was strongest in younger and middle-aged adults. For the 20–34 and 35–50-year age groups, Q4 was significantly associated with dyslipidemia in unadjusted models (ages 20–34: OR 1.89, 95% CI 1.18–3.02, *p* = 0.013; ages 35–50: OR 2.17, 95% CI 1.45–3.25, *p* < 0.001) and after NM adjustment (ages 20–34: OR 1.79, 95% CI 1.11–2.87, *p* = 0.025; ages 35–50: OR 2.28, 95% CI 1.51–3.42, *p* < 0.001). However, across all age groups, the association lost statistical significance when fully adjusted for modifiable (M) factors (BMI, WC, HC, hypertension, and diabetes), with Q4 ORs ranging from 1.11 (95% CI 0.68–1.83) in ages 20–34 to 1.59 (95% CI 0.74–3.40) in ages 65+, none statistically significant.

In fully adjusted models, the association between UA/UL quartiles and dyslipidemia was no longer statistically significant across the total population. Sex- and age-stratified analyses are presented in Table 4 and Table 5. Formal tests of the UA/UL quartile × age interaction were conducted in the unadjusted (*p*-for-interaction = 0.327) and demographic-adjusted models (*p*-for-interaction = 0.369). Neither was statistically significant, indicating that age group does not significantly modify the UA/UL ratio–dyslipidemia association. Age-stratified results are therefore reported as exploratory descriptive observations only.

## 4. Discussion

No other studies to date have reported a direct examination of the association between UA/UL ratio and dyslipidemia. Therefore, we used a nationally representative cross-sectional analysis of U.S. adults to understand the relationship between UA/UL and dyslipidemia. A UA/UL ratio was associated with greater odds of dyslipidemia in unadjusted models and after adjustment for demographic and selected anthropometric or metabolic covariates considered separately. These associations were attenuated and not statistically significant in fully adjusted models that included demographics, adiposity measures (BMI, WC, HC), and comorbidities (hypertension and diabetes). After full adjustment, associations were attenuated to non-significance in both sexes; age-stratified models showed stronger associations in younger and middle-aged adults before full adjustment. The association between UA/UL ratio and dyslipidemia observed in this study is biologically plausible within the Developmental Origins of Health and Disease (DOHaD) framework. Early-life nutritional status is a principal determinant of differential limb segment growth: protein-energy undernutrition during the first two years of life preferentially restricts lower-limb elongation relative to upper-limb growth, producing persistently higher arm-to-leg length ratios in adulthood that serve as fixed anthropometric records of early nutritional deprivation [15,16]. Beyond nutrition, a range of prenatal and postnatal developmental exposures—including intrauterine growth restriction, low birth weight, maternal undernutrition during pregnancy, recurrent childhood infection, and socioeconomic deprivation—have been independently associated with shorter adult leg length and altered skeletal proportions, suggesting that the UA/UL ratio integrates a broad spectrum of early environmental influences into a single fixed adult measurement [18,21]. These developmental exposures are also hypothesized, under the DOHaD framework, to program lasting adaptations in insulin signaling and lipid metabolism that manifest as elevated cardiometabolic risk in adulthood, offering a plausible biological pathway through which a higher UA/UL ratio marks long-term susceptibility to dyslipidemia [16]. Critical knowledge gaps remain, however. The specific developmental windows during which limb growth restriction most strongly predicts adult lipid dysregulation have not been established. Whether the UA/UL ratio captures primarily nutritional deprivation, hormonal disruption, or socioeconomic patterning of early growth cannot be determined from cross-sectional adult data alone. Future research linking prospectively collected early-life growth data to adult UA/UL ratios and lipid profiles would directly test the developmental programming hypothesis and clarify which upstream exposures drive the skeletal proportion–dyslipidemia association observed here. Formal tests of the UA/UL quartile × sex interaction were non-significant in both unadjusted (*p*-for-interaction = 0.984) and adjusted models (*p*-for-interaction = 0.943); These sex-stratified results are therefore reported as exploratory and descriptive only.

The progressive attenuation of the UA/UL ratio–dyslipidemia association across sequential adjustment models warrants careful methodological interpretation. Under the DOHaD framework [16,29], BMI, waist circumference, hip circumference, hypertension, and diabetes are plausible mediators (not confounders) on the causal pathway from developmental programming through the UA/UL ratio to adult dyslipidemia. If this is the case, adjusting for these variables in the regression model constitutes overadjustment: it statistically blocks the very mechanism through which the UA/UL ratio is hypothesized to exert its metabolic effects, biasing the estimated association toward the null. The attenuation observed in Models 3 through 5 is precisely the pattern expected under overadjustment, and the near-null finding in the fully adjusted model is therefore not necessarily evidence of a spurious association. Rather, it is consistent with the UA/UL ratio operating as a developmental marker whose influence on dyslipidemia is substantially mediated through adiposity-related and metabolic pathways. The total causal effect, estimated most cleanly by Model 2, which adjusts only for confirmed confounders, showed significantly elevated odds of dyslipidemia in the highest UA/UL quartile (OR 1.72, 95% CI 1.27–2.33; *p* < 0.001), and this effect persisted after adjustment for non-modifiable demographic factors. The sex-stratified finding, in which neither sex showed a statistically significant association after full adjustment, is consistent with the UA/UL ratio operating through adiposity-mediated pathways that are similarly attenuated in both sexes once modifiable factors are controlled. Formal mediation analysis within longitudinal cohort data would be required to decompose the total effect into direct and adiposity-mediated components, and is identified as a priority for future research. Authors should note that the alternative interpretation, that BMI, WC, and HC are confounders rather than mediators, cannot be ruled out in this cross-sectional design, and both interpretations are acknowledged as limitations.

Conventional anthropometric measures such as BMI, WC, and HC are well-recognized predictors of dyslipidemia [13], as are established metabolic comorbidities such as hypertension and diabetes [30]. In the present analysis, the UA/UL ratio remained significantly associated with dyslipidemia in models that adjusted for these covariates individually, suggesting that fixed skeletal proportions may index aspects of body composition and metabolic risk not fully captured by conventional adiposity or disease-status measures alone [18,19,20,21]. This pattern is consistent with the interpretation that the UA/UL ratio reflects upstream developmental programming that operates through, rather than independently of, established cardiometabolic risk pathways.

Important differences emerged when stratifying the analysis by sex and age group. In sex-stratified analyses, higher UA/UL quartiles were significantly associated with greater odds of dyslipidemia in unadjusted models among both females and males. After full adjustment for modifiable factors and chronic diseases, the association was attenuated and no longer statistically significant in either sex, with point estimates of broadly similar magnitude in females (Q4 OR 1.19, 95% CI 0.86–1.66) and males (Q4 OR 1.42, 95% CI 0.96–2.10). Formal interaction testing confirmed that sex does not significantly modify the UA/UL–dyslipidemia association (*p*-for-interaction = 0.984 unadjusted, 0.943 adjusted). These sex-stratified results are therefore descriptive and exploratory, and the observed pattern should not be interpreted as evidence of genuine effect modification by sex.

Age stratification revealed that the influence of the UA/UL ratio on dyslipidemia was most evident in younger and middle-aged adults and diminished in older adults. In the 20–34 and 35–50-year age groups, those in the highest UA/UL ratio quartile had significantly greater odds of dyslipidemia in models adjusted for non-modifiable factors. This suggests that limb proportion differences may manifest as a risk factor relatively early in adulthood, potentially before chronic conditions fully develop. Younger individuals with disproportionately short legs might identify a subset of the population with developmental risk factors predisposed to abnormal lipid profiles at a comparably early age. These sex and age findings highlight that the UA/UL ratio utility as a risk marker might vary across subpopulations.

### 4.1. Future Directions

Several important future research directions emerge from the present findings. First, prospective longitudinal studies are needed to determine whether the UA/UL ratio independently predicts incident dyslipidemia and downstream cardiovascular events, moving beyond the cross-sectional associations documented here. Second, mediation analyses within cohort data could quantify the extent to which adiposity distribution and insulin resistance pathways explain the UA/UL–dyslipidemia relationship, clarifying whether skeletal limb proportions exert direct metabolic effects or function primarily as markers of upstream developmental programming. Third, the sex-stratified analyses, which showed attenuation of the association in both sexes after full adjustment, warrant replication in prospective cohort data with formal mediation analyses to clarify whether observed sex differences in adjusted estimates reflect genuine biological variation or are attributable to differential statistical power. Fourth, studies linking early-life exposures, including childhood nutritional status, growth trajectories, and socioeconomic environment, to adult UA/UL ratios would directly test whether this metric captures developmental programming of cardiometabolic risk as hypothesized. Fifth, replication in racially, ethnically, and geographically diverse populations is needed, given that limb proportions vary by ancestry and early developmental conditions. Finally, establishing externally validated cutoffs or sex- and age-standardized reference values for the UA/UL ratio would be a prerequisite for evaluating any potential clinical utility of this measure, and should be considered only after prospective evidence of predictive validity is established.

### 4.2. Limitations

The cross-sectional design precludes causal inference. Additionally, UA/UL quartiles were empirically derived as no established clinical cut points exist for this metric; findings should therefore be interpreted as exploratory. Third, despite multivariable adjustment, residual confounding (e.g., childhood nutrition, hormonal milieu, growth timing) cannot be excluded. Finally, measurement constraints (e.g., side-specific limb length substitutions when right-sided measures were not feasible) could introduce nondifferential error that could have biased results. Prospective studies should evaluate whether UA/UL predicts incident dyslipidemia and downstream cardiovascular events, quantify mediation by adiposity distribution and insulin resistance, explore mechanisms underlying sex differences, and assess whether UA/UL improves risk stratification when integrated into multivariable prediction models. Establishing externally validated cut points or standardized z-scores for UA/UL could also facilitate comparability across studies.

## 5. Conclusions

In this nationally representative cross-sectional study, a higher UA/UL was associated with a greater prevalence of dyslipidemia in U.S. adults prior to full adjustment for adiposity and metabolic comorbidities. The attenuation of this association in fully adjusted models suggests that limb proportions may reflect upstream developmental or biological factors that influence established cardiometabolic risk pathways. The UA/UL ratio may serve as a research marker of early-life developmental susceptibility associated with dyslipidemia risk, warranting further investigation in prospective studies before any implications for clinical practice can be drawn. Longitudinal studies are needed to clarify the nature and stability of this observed association and assess its clinical utility.

Sensitivity analyses further showed that the association was driven primarily by hypertriglyceridemia and low HDL-C rather than by elevated LDL-C or total cholesterol, a pattern mechanistically consistent with an insulin resistance-driven metabolic phenotype rather than an atherogenic LDL-driven one.

Our findings extend prior work linking relative limb proportions, especially shorter legs, to adverse cardiometabolic profiles, including insulin resistance and lipid abnormalities. Multiple studies have reported that leg-to-trunk proportions relate to metabolic risk beyond overall adiposity, supporting the biological plausibility that fixed skeletal proportions capture early-life growth and developmental influences relevant to lipid metabolism. Additionally, the disappearance of the association in fully adjusted models suggests that UA/UL may index upstream factors that largely operate through established adiposity and metabolic pathways. Thus, UA/UL appears associated with dyslipidemia but is not independent of conventional risk factors when modeled simultaneously in the overall sample. Formal interaction testing (UA/UL quartile × sex: *p*-for-interaction = 0.943) confirmed that sex does not significantly modify the UA/UL–dyslipidemia association. The observed pattern does not reflect genuine effect modification and likely reflects chance variation from multiple subgroup comparisons. Replication in independent datasets is needed before mechanistic conclusions can be drawn.

## Figures and Tables

**Table 1:** Weighted characteristics of U.S. adults aged ≥20 years by dyslipidemia status, NHANES 2017–March 2020.

Characteristic	No Dyslipidemia, %	Dyslipidemia, %	*p* Value
**Age group, years**			
20–34 (*n* = 1680)	49.2	50.8	
35–50 (*n* = 1894)	30.3	69.7	<0.001
51–65 (*n* = 2214)	19.1	80.9	
≥65 (*n* = 1781)	13.8	86.2	
**Sex**			
Male (*n* = 3731)	29.5	70.5	0.674
Female (*n* = 3838)	28.7	71.3	
**Race/ethnicity**			
Mexican American (*n* = 902)	29.1	70.9	
Other Hispanic (*n* = 777)	29.6	70.4	<0.001
Non-Hispanic White (*n* = 2693)	27.6	72.4	
Non-Hispanic Black (*n* = 1930)	38.9	61.1	
Other/Multiracial (*n* = 1267)	27.8	72.2	
**BMI category**			
Underweight (*n* = 101)	53.6	46.4	
Normal weight (*n* = 1809)	47.4	52.6	<0.001
Overweight (*n* = 2446)	26.2	73.8	
Obesity (*n* = 3194)	19.5	80.5	
**Waist circumference category**			
Normal (*n* = 1633)	50.1	49.9	<0.001
At Risk (*n* = 1342)	32.8	67.2	
Elevated (*n* = 4541)	20.2	79.8	<0.001
**Hypertension status**			
No (*n* = 2901)	40.5	59.5	<0.001
Yes (*n* = 4296)	17.8	82.2	
**Diabetes status**			
No (*n* = 5981)	32.5	67.5	<0.001
Yes (*n* = 1525)	11.0	89.0	
**Hip circumference quartile**			
Q1 (*n* = 1464)	42.1	57.9	
Q2 (*n* = 1949)	33.1	66.8	<0.001
Q3 (*n* = 2056)	25.8	74.2	
Q4 (*n* = 2060)	20.6	79.4	
**Upper arm to upper leg ratio quartile**			
Q1 (*n* = 1329)	41.6	58.4	
Q2 (*n* = 1699)	34.3	65.7	<0.001
Q3 (*n* = 2076)	27.3	72.7	
Q4 (*n* = 2465)	18.7	81.3	

Missing (unweighted): BMI (*n* = 19), WC (*n* = 53), hypertension (*n* = 372), diabetes (*n* = 63), HC (*n* = 40). Percentages may not sum to 100.0 due to rounding. Values are weighted column percentages; unweighted sample sizes (*n*) are shown. *p*-values are from Rao–Scott chi-square tests.

**Table 2:** Weighted characteristics of U.S. adults aged 20 years or older by upper arm to upper leg ratio quartile (NHANES 2017 to March 2020).

Characteristic	Overall %	Q1% (*n* = 1329)	Q2% (*n* = 1699)	Q3% (*n* = 2076)	Q4% (*n* = 2465)	*p* Value
**Age group, years**						
20–34 (*n* = 1680)	26.9	45.1	35.2	25.3	10.3	<0.001
35–50 (*n* = 1894)	26.2	31.0	31.1	24.5	20.8	
51–65 (*n* = 2214)	27.2	17.4	23.3	30.8	33.3	
≥65 (*n* = 1781)	19.6	6.6	10.3	19.4	35.6	
**Sex**						
Male (*n* = 3731)	49.0	56.4	51.6	51.0	40.3	<0.001
Female (*n* = 3838)	51.0	43.6	48.4	49.0	59.7	
**Race/ethnicity**						<0.001
Mexican American (*n* = 902)	8.4	8.2	8.2	8.4	8.5	
Other Hispanic (*n* = 777)	7.4	7.8	7.7	6.7	7.7	
Non-Hispanic White (*n* = 2693)	63.9	54.0	62.2	66.6	68.7	
Non-Hispanic Black (*n* = 1930)	10.7	19.3	10.7	8.2	7.5	
Other/Multiracial (*n* = 1267)	9.7	10.6	11.1	10.1	7.6	
**BMI category**						
Underweight (*n* = 101)	1.4	2.4	2.2	1.1	0.2	
Normal weight (*n* = 1809)	24.9	32.5	30.8	26.4	14.0	<0.001
Overweight (*n* = 2446)	32.1	33.4	33.7	33.1	28.9	
Obesity (*n* = 3194)	41.6	31.7	33.3	39.4	56.8	
**Waist circumference category**						
Normal	22.6	35.9	28.4	22.0	10.2	
Increased risk	17.8	20.4	20.8	19.4	12.3	<0.001
Substantially increased risk	59.5	43.6	50.8	58.7	77.5	
**Hypertension status**						
No (*n* = 2901)	47.3	63.1	55.9	46.5	31.4	<0.001
Yes (*n* = 4296)	52.7	36.9	44.1	53.5	68.6	
**Diabetes status**						<0.001
No (*n* = 5981)	85.0	93.5	90.8	85.9	74.1	
Yes (*n* = 1525)	15.0	6.5	9.2	14.1	25.9	
**Hip circumference quartile**						
Q1 (lowest) (*n* = 1464)	17.6	21.3	22.0	18.0	11.2	
Q2 (*n* = 1949)	26.0	28.8	28.0	27.8	20.9	<0.001
Q3 (*n* = 2056)	28.9	28.2	28.9	30.6	27.8	
Q4 (highest) (*n* = 2060)	27.5	21.8	21.1	23.6	40.0	
**Dyslipidemia status**						
No (*n* = 2084)	29.1	41.6	34.3	27.3	18.7	<0.001
Yes (*n* = 5485)	70.9	58.4	65.7	72.7	81.3	

Note: Percentages are survey weighted; sample sizes (*n*) are unweighted. *p*-values are from the Rao Scott chi square test. Categories may not sum to the full sample due to missing data (BMI missing = 19, WC missing = 53, hypertension missing = 372, diabetes missing = 63, HC missing = 40). WC categories were based on WHO risk thresholds (normal, increased risk, substantially increased risk) using sex specific cut points.

**Table 3:** Survey-weighted odds ratios (OR) and 95% confidence intervals (CI) for the association between UA/UL ratio quartile and dyslipidemia prevalence, NHANES 2017–March 2020.

	Model 1 Crude	Model 2 Demographic Adjusted *	Model 3 Anthropometric Adjusted ^†^	Model 4 Metabolic Adjusted ^‡^	Model 5 Fully Adjusted ^§^
*n*	7538	7538	7401 ^1^	7044	7401
UA/UL ratio quartile (Q1 = lowest; reference)					
Q1 (<0.898; reference)	Reference	Reference	Reference	Reference	Reference
Q2 (0.898–0.942)	**1.36 (1.11–1.66)** ***p* = 0.003**	**1.18 (0.90–1.53)** ***p* = 0.225**	**1.25 (1.01–1.55)** ***p* = 0.043**	**1.27 (1.00–1.60)** ***p* = 0.047**	1.03 (0.78–1.37) *p* = 0.822
Q3 (0.942–0.993)	**1.89 (1.52–2.35)** ***p* < 0.001**	**1.37 (1.05–1.78)** ***p* = 0.020**	**1.56 (1.23–1.97)** ***p* < 0.001**	**1.55 (1.19–2.00)** ***p* = 0.001**	1.03 (0.77–1.40) *p* = 0.821
Q4 (≥0.993; highest)	**3.10 (2.48–3.86)** ***p* < 0.001**	**1.72 (1.27–2.33)** ***p* < 0.001**	**1.97 (1.58–2.46)** ***p* < 0.001**	**2.01 (1.53–2.64)** ***p* < 0.001**	0.92 (0.65–1.32) *p* = 0.654
Adjustment covariates—Demographic					
Age group (vs. 20–34 years)	—	Adjusted	—	—	Adjusted
Sex	—	Adjusted	—	—	Adjusted
Race/ethnicity	—	Adjusted	—	—	Adjusted
Adjustment covariates—Anthropometric					
BMI (per kg/m^2^)	—	—	Adjusted	—	Adjusted
Waist circumference (per cm)	—	—	Adjusted	—	Adjusted
Hip circumference (per cm)	—	—	Adjusted	—	Adjusted
Adjustment covariates—Metabolic/Clinical					
Hypertension (yes vs. no)	—	—	—	Adjusted	Adjusted
Diabetes (yes vs. no)	—	—	—	Adjusted	Adjusted

**Bold** = statistically significant (*p* < 0.05). Reference category: Q1 (UA/UL < 0.898). Survey design accounted for using WTMECPRP weights, SDMVPSU clusters, and SDMVSTRA strata. * Model 2 adjusted for age group, sex, and race/ethnicity. ^†^ Model 3 adjusted for BMI, waist circumference, and hip circumference. ^‡^ Model 4 adjusted for hypertension and diabetes. ^§^ Model 5 adjusted for all demographic, anthropometric, and metabolic/clinical covariates. ^1^ Model 3 sample size reduced because of missing values for one or more anthropometric variables. Abbreviations: BMI, body mass index; CI, confidence interval; HC, hip circumference; OR, odds ratio; UA/UL, upper arm-to-upper leg length ratio; WC, waist circumference.

**Table 4:** Survey-weighted odds ratios (OR) and 95% confidence intervals (CI) for the association between UA/UL ratio quartile and dyslipidemia, stratified by sex, NHANES 2017–March 2020.

UA/UL Quartile	Female Unadjusted (*n* = 3838)	Female Adjusted (*n* = 3546)	Male Unadjusted (*n* = 3731)	Male Adjusted (*n* = 3517)
	OR (95% CI)	OR (95% CI)	OR (95% CI)	OR (95% CI)
Q1 (<0.898; reference)	Reference	Reference	Reference	Reference
Q2 (0.898–0.942)	1.34 (1.08–1.66) *p* = 0.012	1.27 (0.97–1.67) *p* = 0.097	1.38 (0.99–1.93) *p* = 0.067	0.99 (0.69–1.41) *p* = 0.939
Q3 (0.942–0.993)	1.92 (1.39–2.64) *p* < 0.001	1.30 (0.92–1.84) *p* = 0.151	1.88 (1.39–2.54) *p* < 0.001	1.19 (0.83–1.70) *p* = 0.362
Q4 (≥0.993; highest)	3.07 (2.39–3.95) *p* < 0.001	1.19 (0.86–1.66) *p* = 0.307	3.19 (2.29–4.46) *p* < 0.001	1.42 (0.96–2.10) *p* = 0.091
*p*-for-interaction	Unadjusted: 0.984 | Adjusted: 0.943		See female columns (UA/UL × sex interaction)	

Note: Adjusted for Modifiable variables and chronic disease (e.g., BMI, WC, HC, HTN, and Diabetes).

**Table 5:** Survey-weighted odds ratios (OR) and 95% confidence intervals (CI) for the association between UA/UL ratio quartile and dyslipidemia, stratified by age group, NHANES 2017–March 2020.

Age Group	Model	Q1 (Reference)	Q2 OR (95% CI) 0.898–0.942	Q3 OR (95% CI) 0.942–0.993	Q4 OR (95% CI) ≥ 0.993
20–34 years	Unadjusted *n* = 1680	Ref	0.98 (0.72–1.34) *p* = 0.900	1.34 (0.90–1.98) *p* = 0.163	1.89 (1.18–3.02) *p* = 0.013
	NM Adjusted *n* = 1680	Ref	0.97 (0.69–1.34) *p* = 0.840	1.30 (0.87–1.93) *p* = 0.211	1.79 (1.11–2.87) *p* = 0.025
	M Adjusted *n* = 1549	Ref	0.88 (0.58–1.34) *p* = 0.563	1.07 (0.63–1.83) *p* = 0.791	1.11 (0.68–1.83) *p* = 0.680
35–50 years	Unadjusted *n* = 1894	Ref	1.37 (0.85–2.22) *p* = 0.208	1.35 (0.89–2.04) *p* = 0.174	2.17 (1.45–3.25) *p* = 0.001
	NM Adjusted *n* = 1894	Ref	1.36 (0.84–2.18) *p* = 0.217	1.34 (0.88–2.06) *p* = 0.190	2.27 (1.51–3.42) *p* = 0.001
	M Adjusted *n* = 1758	Ref	1.29 (0.76–2.19) *p* = 0.352	1.06 (0.65–1.74) *p* = 0.823	1.32 (0.84–2.06) *p* = 0.240
51–65 years	Unadjusted *n* = 2214	Ref	1.60 (0.93–2.76) *p* = 0.105	1.59 (0.82–3.07) *p* = 0.181	1.70 (0.88–3.27) *p* = 0.124
	NM Adjusted *n* = 2214	Ref	1.58 (0.90–2.77) *p* = 0.121	1.53 (0.79–2.98) *p* = 0.216	1.61 (0.86–3.00) *p* = 0.147
	M Adjusted *n* = 2084	Ref	1.54 (0.89–2.68) *p* = 0.139	1.39 (0.70–2.76) *p* = 0.351	1.20 (0.61–2.38) *p* = 0.603
65+ years	Unadjusted *n* = 1781	Ref	1.44 (0.70–2.94) *p* = 0.331	1.95 (0.77–4.96) *p* = 0.173	2.02 (0.92–4.42) *p* = 0.092
	NM Adjusted *n* = 1781	Ref	1.32 (0.62–2.82) *p* = 0.483	1.72 (0.65–4.54) *p* = 0.287	1.64 (0.71–3.75) *p* = 0.254
	M Adjusted *n* = 1672	Ref	1.29 (0.63–2.63) *p* = 0.487	1.90 (0.74–4.90) *p* = 0.195	1.58 (0.74–3.40) *p* = 0.248

*p*-for-interaction (UA/UL quartile × age group): unadjusted *p* = 0.327; NM-adjusted *p* = 0.369. Neither is statistically significant; age-stratified results are exploratory and descriptive only. Note: *p*-value < 0.05. NM = Adjusted for Non-Modifiable variables (e.g., Sex, Race). M = Adjusted for Modifiable variables (e.g., BMI, WC, HC, and Diabetes).

## Data Availability

The data used in this study are publicly available from the National Health and Nutrition Examination Survey (NHANES) website at https://wwwn.cdc.gov/nchs/nhanes/ (accessed on 24 March 2026). All data files analyzed are publicly accessible without restriction.

## Supplementary Materials

The following supporting information can be downloaded at: https://www.mdpi.com/article/10.3390/ijerph23050662/s1, Table S1: Comparison of baseline characteristics between included and excluded participants, NHANES 2017–March 2020; Table S2: Component-specific sensitivity analyses: survey-weighted logistic regression for individual lipid outcome components, NHANES 2017–March 2020.
