# Supplementary material for: Upper Arm to Upper Leg Length Ratio and Dyslipidemia: A Novel Application of a Fixed Skeletal Proportion Metric in a Nationally Representative U.S. Sample
Source: Int J Environ Res Public Health. 2026 May 16;23(5):662. doi: 10.3390/ijerph23050662 (PMC13207109; doi:10.3390/ijerph23050662)
Supplement: Supplementary file 1 [file ijerph-23-00662-s001.zip › ijerph-4250642-supplementary.pdf]

Supplementary Table S1. Comparison of baseline characteristics between included and excluded participants, NHANES 2017–March 2020.

Characteristic	Included (n = 7,569)	Excluded[a] (n = 7,991)	p-value
**Continuous variables, mean (SE)**			
Age (years)	51.1 (0.2)	17.3 (0.2)	<0.001
BMI (kg/m²)	29.8 (0.1)	22.4 (0.1)	<0.001
Waist circumference (cm)	100.8 (0.2)	73.1 (0.3)	<0.001
Hip circumference (cm)	107.2 (0.2)	100.9 (0.3)	<0.001
Upper arm length (cm)	37.6 (0.0)	28.9 (0.1)	<0.001
Upper leg length (cm)	39.1 (0.0)	38.0 (0.1)	<0.001
**Sex, n (%)**			
Male	3,731 (49.3)	3,990 (49.9)	0.347
Female	3,838 (50.7)	4,001 (50.1)	
**Race/ethnicity, n (%)**			
Mexican American	902 (11.9)	1,088 (13.6)	<0.001
Other Hispanic	777 (10.3)	767 (9.6)	
Non-Hispanic White	2,693 (35.6)	2,578 (32.3)	
Non-Hispanic Black	1,930 (25.5)	2,168 (27.1)	
Other/Multiracial	1,267 (16.7)	1,390 (17.4)	
**BMI category, n (%)[b]**			
Underweight (<18.5)	101 (1.3)	2,299 (41.2)	<0.001
Normal weight (18.5–24.9)	1,809 (24.0)	1,760 (31.5)	
Overweight (25.0–29.9)	2,446 (32.4)	729 (13.0)	
Obesity (≥30)	3,194 (42.3)	799 (14.3)	
**Hypertension status, n (%)[c]**			
Non-hypertensive	2,901 (40.3)	2,888 (77.3)	<0.001
Hypertensive	4,296 (59.7)	846 (22.7)	
**Diabetes status, n (%)[d]**			
Non-diabetic	5,981 (79.7)	2,102 (87.9)	<0.001
Diabetic	1,525 (20.3)	288 (12.1)	
**Dyslipidemia status, n (%)[e]**			
No dyslipidemia	2,084 (27.5)	2,140 (58.8)	<0.001

Characteristic	Included (n = 7,569)	Excluded[a] (n = 7,991)	p-value
Dyslipidemia	5,485 (72.5)	1,500 (41.2)	

Abbreviations: BMI, body mass index; NHANES, National Health and Nutrition Examination Survey; SE, standard error.

[a] Excluded participants comprise three sequential exclusion groups: age <20 years (n = 6,328; 79.2% of all excluded); pregnant at examination (n = 87; 1.1%); and missing dyslipidemia classification or UA/UL ratio (n = 1,576; 19.7%). The large difference in age and anthropometric measures between included and excluded participants is primarily driven by the age exclusion, as children and adolescents differ substantially from adults on all anthropometric measures.

[b] BMI data missing for n = 19 (included) and n = 2,404 (excluded, predominantly the age-excluded subgroup <20 years with non-standard BMI reference ranges).

[c] Hypertension data missing for n = 372 (included) and n = 4,257 (excluded). Percentages calculated among those with non-missing data.

[d] Diabetes data missing for n = 63 (included) and n = 5,601 (excluded). Percentages calculated among those with non-missing data.

[e] Dyslipidemia data missing for n = 0 (included by definition) and n = 4,351 (excluded). Percentages calculated among those with non-missing data.

p-values for continuous variables are from independent samples t-tests comparing unweighted means; p-values for categorical variables are from chi-square tests. All counts are unweighted. Caution is warranted in interpreting these comparisons given that the excluded group is predominantly composed of children and adolescents rather than adults meeting other exclusion criteria.

Supplementary Table S2. Component-specific sensitivity analyses: survey-weighted logistic regression for individual lipid outcome components, NHANES 2017–March 2020.

Lipid Outcome	Criterion	n	Overall p	Quartile	OR	95% CI	p-value	Sig.
**Hypertriglyceridemia**	TG ≥150 mg/dL	7,569	**p < 0.001**	Q1	*Reference*	—	—	
				Q2	1.02	0.75 – 1.38	p = 0.892	
				Q3	**1.41**	1.07 – 1.86	**p = 0.018**	*
				Q4	**1.84**	1.31 – 2.60	**p = 0.001**	**
**Low HDL-C**	Sex-specific	7,569	**p < 0.001**	Q1	*Reference*	—	—	
				Q2	1.02	0.79 – 1.33	p = 0.862	
				Q3	1.09	0.80 – 1.47	p = 0.577	
				Q4	**1.97**	1.58 – 2.46	**p < 0.001**	***
**Elevated total cholesterol**	TC ≥200 mg/dL	7,569	p = 0.369	Q1	*Reference*	—	—	
				Q2	1.08	0.87 – 1.33	p = 0.481	
				Q3	0.98	0.73 – 1.32	p = 0.896	
				Q4	0.87	0.65 – 1.17	p = 0.355	
**Elevated LDL-C**	LDL-C ≥130 mg/dL	7,399[a]	p = 0.623	Q1	*Reference*	—	—	
				Q2	1.07	0.85 – 1.33	p = 0.560	
				Q3	0.91	0.68 – 1.20	p = 0.478	
				Q4	0.91	0.68 – 1.23	p = 0.540	
**Lipid-lowering medication use**	Current use	6,021[b]	**p = 0.005**	Q1	*Reference*	—	—	
				Q2	1.36	0.99 – 1.86	p = 0.058	
				Q3	**1.63**	1.14 – 2.33	**p = 0.009**	**
				Q4	**2.04**	1.42 – 2.95	**p < 0.001**	***

Q1 = reference category (UA/UL < 0.898). **Bold** = statistically significant (p < 0.05). *** p < 0.001 ** p < 0.01 * p < 0.05

All models adjusted for age group, sex, and race/ethnicity (Model 2 equivalent). Survey design accounted for using WTMECPRP weights, SDMVPSU clusters, and SDMVSTRA strata.

[a] LDL-C model: n = 7,399 (170 excluded; triglycerides ≥400 mg/dL renders Friedewald equation invalid).

[b] Lipid-lowering medication model: n = 6,021 (1,548 excluded; missing BPQ090D). The dose-response pattern likely reflects age confounding and should be interpreted with caution.

Abbreviations: CI, confidence interval; HDL-C, high-density lipoprotein cholesterol; LDL-C, low-density lipoprotein cholesterol; OR, odds ratio; TC, total cholesterol; TG, triglycerides; UA/UL, upper arm-to-upper leg length ra
